# Young Adults With Acute Myocardial Infarction in Morocco: An Emerging Cardiovascular Challenge

**DOI:** 10.7759/cureus.105054

**Published:** 2026-03-11

**Authors:** Soufiane Touiti, Nada Fennich, Jamila Zarzur, Latifa Oukerraj, Cherti Mohamed

**Affiliations:** 1 Department of Clinical Cardiology, Cardiology B Hospital, Mohammed V University of Rabat, Rabat, MAR; 2 Department of Cardiac Catheterization, Cardiology B Hospital, Mohammed V University of Rabat, Rabat, MAR

**Keywords:** acute st-elevation myocardial infarction, behavioral cardiovascular risk factors, coronary artery angiography, tobacco and cannabis use, young adults

## Abstract

Acute myocardial infarction (AMI) in young adults - commonly defined as occurring before the age of 50 years - represents a distinct clinical entity with specific epidemiological and angiographic characteristics. Although its incidence is increasing worldwide, data from North African countries remain limited, and behavioral risk factors, particularly tobacco and cannabis use, appear to play a major role in premature coronary events. We conducted a retrospective single-center descriptive study including patients aged ≤49 years admitted with ST-segment elevation myocardial infarction (STEMI) between January and December 2025 at a tertiary university hospital in Morocco.

A total of 62 patients were included, with a mean age of 41 ± 7 years and a predominance of males (77%). Smoking was the most prevalent cardiovascular risk factor (64.5%), followed by cannabis use (27.4%). Coronary angiography revealed predominantly single-vessel disease (77.4%), with the left anterior descending artery as the most frequent culprit vessel (64.7%). Myocardial infarction with non-obstructive coronary arteries (MINOCA) was identified in 9.7% of patients. Primary percutaneous coronary intervention was performed in 61.3% of cases, and successful reperfusion was achieved in 74.2%. In-hospital complications included left ventricular systolic dysfunction (23%) and cardiogenic shock (9.7%), while in-hospital mortality remained low (1.6%).

AMI in young adults in Morocco is largely driven by modifiable behavioral risk factors and is characterized by predominantly single-vessel coronary disease with generally favorable short-term outcomes. These findings highlight the importance of targeted prevention strategies and optimized acute management to reduce the burden of premature myocardial infarction in this population.

## Introduction

Acute myocardial infarction (AMI) in young adults, generally defined as occurring before the age of 50 years, represents a distinct clinical entity accounting for approximately 5-10% of all myocardial infarctions [1,2]. Although less frequent than in older populations, its incidence has shown a concerning upward trend worldwide over the past decades, particularly among young men [2]. Unlike AMI in older patients, which is predominantly driven by long-standing atherosclerotic disease and metabolic risk factors, premature AMI is more often associated with behavioral and lifestyle-related risk factors, including cigarette smoking, recreational drug use - especially cannabis - and, to a lesser extent, dyslipidemia or congenital coronary abnormalities [3].

Young patients with AMI typically present with a different coronary phenotype, characterized by a predominance of single-vessel disease, a lower overall atherosclerotic burden, and a higher proportion of non-obstructive coronary syndromes such as myocardial infarction with non-obstructive coronary arteries (MINOCA) [3]. These features reflect a broader spectrum of underlying mechanisms, including plaque erosion, transient thrombosis, coronary vasospasm, spontaneous coronary artery dissection (SCAD), or embolic phenomena, which pose important diagnostic and therapeutic challenges.

In low- and middle-income countries, including those in North Africa, data on AMI in young adults remain rare despite a high prevalence of tobacco use and increasing cannabis consumption among younger populations. In Morocco, in particular, national epidemiological data describing the clinical profile, coronary anatomy, management strategies, and short-term outcomes of young patients presenting with AMI are limited. This lack of local evidence hampers the development of targeted prevention strategies and optimized care pathways adapted to this high-risk but potentially preventable population.

Therefore, the present study aimed to analyze the epidemiological characteristics, cardiovascular risk factors, coronary angiographic patterns, reperfusion strategies, and in-hospital outcomes of young patients admitted with ST-segment elevation myocardial infarction (STEMI) in a Moroccan university hospital. By highlighting local specificities and comparing our findings with international data, we seek to identify opportunities for improving primary prevention, early diagnosis, and acute management of AMI in young adults.

## Materials and methods

This was a retrospective descriptive observational single-center study conducted in the Cardiology B Department of Ibn Sina University Hospital, Rabat, Morocco. The study period extended from January 2025 to December 2025. Medical records of young patients admitted for AMI during this period were retrospectively reviewed using a standardized data collection form.

Eligible patients were all consecutive individuals aged ≤49 years who were admitted with a confirmed diagnosis of STEMI during the study period. STEMI was diagnosed according to the criteria defined by the European Society of Cardiology (ESC) guidelines and the Fourth Universal Definition of Myocardial Infarction, based on persistent ST-segment elevation in at least two contiguous leads in the appropriate clinical context, with subsequent elevation of cardiac biomarkers [4]. Patients older than 49 years, those not fulfilling STEMI diagnostic criteria, patients primarily managed in other hospital departments, and cases with incomplete or non-exploitable medical records were excluded. During the study period, all consecutive eligible patients were screened. A total of 73 patients were initially identified; 11 were excluded due to incomplete or missing data, resulting in a final cohort of 62 patients.

Cardiovascular risk factors were recorded as binary variables (present/absent) based on documented medical history at admission. Smoking was defined as active tobacco use documented in the medical record at the time of hospitalization. Hypertension was defined as a prior diagnosis of hypertension or ongoing antihypertensive treatment. Diabetes mellitus was defined as a documented diagnosis of diabetes or use of glucose-lowering therapy. Dyslipidemia was defined as a documented history of lipid disorder or ongoing lipid-lowering treatment. Obesity was defined as a body mass index (BMI) ≥30 kg/m² when available in the medical record. Cannabis and alcohol use were recorded based on self-reported consumption documented in the admission medical file.

The collected data comprised demographic characteristics, cardiovascular risk factors, and clinical presentation, including electrocardiographic findings and infarct territory. Paraclinical data focused on coronary angiographic findings, including the number of diseased vessels, identification of the culprit artery, and angiographic patterns. Therapeutic management was recorded, including reperfusion strategies (primary percutaneous coronary intervention (PCI), thrombolysis, surgical revascularization, or no reperfusion).

Successful reperfusion was defined as restoration of Thrombolysis in Myocardial Infarction (TIMI) flow grade 2 or 3 after primary PCI, or, in patients receiving thrombolysis, ≥50% resolution of ST-segment elevation within 60-90 minutes when documented. Left ventricular systolic dysfunction was defined as a left ventricular ejection fraction (LVEF) <50% on transthoracic echocardiography performed during hospitalization.

In-hospital outcomes, including left ventricular systolic dysfunction, cardiogenic shock, rhythm disturbances, and in-hospital mortality, were analyzed.

Data entry and management were performed using Microsoft Excel (Redmond, WA, USA). Statistical analyses were conducted using Microsoft Excel and Jamovi software. Descriptive statistics were used to summarize the data. Continuous variables were assessed for normality using the Shapiro-Wilk test and were expressed as mean ± standard deviation or median (interquartile range) when appropriate. Categorical variables were presented as frequencies and percentages with corresponding 95% confidence intervals. Given the descriptive nature of the study, no multivariable analyses were performed.

## Results

A total of 62 patients aged ≤49 years were included in the analysis. The mean age was 41 ± 7 years. There was a marked male predominance, with 48 men (77%) and 14 women (23%).

The cardiovascular risk profile was dominated by behavioral risk factors (Table 1). Cigarette smoking was the most prevalent risk factor, observed in 40 patients (64.5%), followed by cannabis use in 17 patients (27.4%). Diabetes mellitus was present in 18 patients (29%), hypertension in nine (14.5%), and dyslipidemia in 11 (17.7%). Other risk factors included obesity in six patients (9.7%), family history of coronary artery disease in five (8.1%), alcohol consumption in five (8.1%), and prior coronary artery disease in four (6.5%). Tobacco and cannabis use were predominantly observed among male patients.

**Table 1:** Cardiovascular Risk Factors in Young Patients With ST-Segment Elevation Myocardial Infarction (STEMI) Sex-specific percentages are presented as proportions within each sex category (48 men and 14 women). CAD: coronary artery disease

Risk factors	Number n (%)	Male n (%) (n=48)	Female n (%) (n=14)
Smoking	40 (64.5%)	37 (77.1%)	3 (21.4%)
Cannabis use	17 (27.4%)	15 (31.3%)	2 (14.3%)
Hypertension	9 (14.5%)	6 (12.5%)	3 (21.4%)
Diabetes mellitus	18 (29%)	13 (27.1%)	5 (35.7%)
Dyslipidemia	11 (17.7%)	7 (14.6%)	4 (28.6%)
Family history of CAD	5 (8.1%)	3 (6.3%)	2 (14.3%)
Obesity	6 (9.7%)	4 (8.3%)	2 (14.3%)
Prior coronary artery disease	4 (6.5%)	2 (4.2%)	2 (14.3%)
Alcohol use	5 (8.1%)	5 (10.4%)	0 (0%)

Chest pain was the predominant presenting symptom. Pain intensity assessed using a visual analog scale was predominantly moderate in 44 patients (71%), followed by severe in 17 (27.4%) and mild in one (1.6%).

Electrocardiographic analysis revealed pathological Q waves suggestive of myocardial necrosis in 41 patients (66%). Infarct localization was most commonly anterior in 16 patients (25%), followed by inferior in 14 (23%), antero-septo-apical in 12 (20%), lateral in 12 (19%), and basal in eight (13%).

Coronary angiography showed single-vessel disease in 48 patients (77.4%), bi-vessel disease in six (9.7%), and tri-vessel disease in two (3.2%). MINOCA was identified in six patients (9.7%). Among patients with obstructive coronary artery disease (n = 56), the left anterior descending artery was the most frequently identified culprit vessel in 36 patients (64.7%), followed by the left circumflex artery in 12 (21.2%) and the right coronary artery in eight (14.1%).

Regarding therapeutic management, primary percutaneous coronary intervention was performed in 38 patients (61.3%), thrombolytic therapy in 14 (22.6%), and surgical revascularization in four (6.5%). No reperfusion was performed in six patients (9.6%) due to delayed presentation or contraindications. Overall, successful reperfusion was achieved in 46 patients (74.2%). Unsuccessful reperfusion was mainly related to persistent impaired coronary flow (TIMI 0-1), no-reflow phenomenon after primary PCI, failure of thrombolysis with absent ST-segment resolution, or delayed presentation beyond the recommended reperfusion window.

The mean length of hospital stay was three days (range 2-5 days). During hospitalization, left ventricular systolic dysfunction occurred in 14 patients (23%), cardiogenic shock in six (9.7%), and ischemic stroke in three (4.8%). Rhythm disturbances were recorded when documented in the medical file but were not systematically subclassified due to the retrospective nature of the study. Overall survival was high, with 61 patients (98.4%) discharged alive, corresponding to an in-hospital mortality rate of one patient (1.6%).

## Discussion

AMI in young adults represents a growing and distinct clinical entity, characterized by specific epidemiological, clinical, and angiographic features that clearly differentiate it from myocardial infarction occurring in older populations [2,3]. In the present study, we observed a strong male predominance and a mean age in the early 40s, findings consistent with contemporary international registries and reflecting early exposure of young men to high-risk behaviors [1,4]. The marked male predominance likely reflects both higher rates of tobacco and cannabis use and a potential protective effect of female sex hormones in premenopausal women [5].

One of the most striking findings of our study is the overwhelming predominance of behavioral cardiovascular risk factors, particularly cigarette smoking and cannabis use. Tobacco use affected nearly two-thirds of patients, reinforcing its central role in premature coronary artery disease through endothelial dysfunction, enhanced platelet activation, and accelerated atherothrombosis [6]. Cannabis use was also highly prevalent, affecting more than one quarter of patients, and has been increasingly implicated in the pathogenesis of acute coronary syndromes in young adults [7]. Experimental and clinical data suggest that cannabis may promote coronary vasospasm, increase myocardial oxygen demand, and induce prothrombotic states, thereby acting as a potent trigger for AMI in susceptible individuals [8,9]. In regions such as Morocco, where cannabis consumption remains relatively common among young adults, these findings underscore the need for stronger public health policies addressing both tobacco and cannabis use as major cardiovascular risk factors.

From an angiographic standpoint, our results confirm the predominance of single-vessel coronary disease in young patients with STEMI, with a marked involvement of the left anterior descending artery. This pattern is consistent with previous studies reporting less diffuse atherosclerosis and more localized culprit lesions in younger individuals [3,10]. Such angiographic features are often associated with plaque erosion or rupture in otherwise minimally diseased coronary arteries, as well as with transient thrombotic occlusions [11]. This limited coronary involvement likely contributes to the relatively favorable short-term prognosis observed in young patients, including lower in-hospital mortality rates compared with older populations.

Nevertheless, the identification of MINOCA in nearly 10% of patients highlights the heterogeneity of mechanisms underlying myocardial infarction in this age group. MINOCA represents a diagnostic and therapeutic challenge, as it encompasses a wide range of pathophysiological processes, including coronary vasospasm, SCAD, coronary embolism, and microvascular dysfunction [12-14]. In young patients, particularly women, these mechanisms may be more prevalent than classic atherothrombotic disease [13]. The limited access to advanced diagnostic tools such as early cardiac magnetic resonance imaging, intracoronary imaging with optical coherence tomography or intravascular ultrasound, and vasoreactivity testing may lead to underdiagnosis or misclassification of these entities in routine clinical practice, especially in low- and middle-income countries [12].

Although short-term outcomes in our cohort were generally favorable, with a high rate of successful reperfusion and low in-hospital mortality, a substantial proportion of patients developed left ventricular systolic dysfunction or cardiogenic shock. These complications carry important prognostic implications and emphasize that young age does not confer protection against severe manifestations of myocardial infarction [15]. Moreover, the long-term prognosis of these patients remains a major concern, as continued exposure to modifiable risk factors such as tobacco and cannabis is associated with recurrent ischemic events, progressive coronary disease, and impaired quality of life [9,16].

Taken together, our findings highlight the urgent need for comprehensive prevention and management strategies tailored to young adults with myocardial infarction. Beyond optimizing acute reperfusion strategies and reducing system delays, particular emphasis should be placed on aggressive secondary prevention, including structured smoking and cannabis cessation programs, early initiation of evidence-based pharmacotherapy, and access to age-adapted cardiac rehabilitation [15,17]. In addition, the implementation of standardized diagnostic pathways for MINOCA and other non-atherosclerotic causes of myocardial infarction may improve etiological diagnosis and guide personalized management in this challenging population [12].

Limitations

This study has several limitations that should be acknowledged. First, its retrospective and single-center design limits the ability to establish causal relationships and may reduce the generalizability of the findings to other populations or healthcare settings. Second, the relatively small sample size may have limited the statistical power to detect associations between risk factors and clinical outcomes, particularly in subgroup analyses.

Third, data collection relied on retrospective medical records, which may be subject to incomplete or missing information, especially regarding lifestyle factors such as the duration, intensity, and timing of tobacco and cannabis use. Fourth, advanced diagnostic investigations, including early cardiac magnetic resonance imaging, intracoronary imaging (optical coherence tomography/intravascular ultrasound), and systematic vasoreactivity testing, were not routinely available. As a result, some non-atherosclerotic mechanisms of myocardial infarction, particularly within the MINOCA spectrum, may have been underdiagnosed or potentially misclassified.

Finally, this study focused on in-hospital outcomes, and no long-term follow-up data were available. Therefore, conclusions regarding recurrent events, long-term mortality, and adherence to secondary prevention strategies could not be assessed.

## Conclusions

Myocardial infarction in young adults represents an emerging and clinically distinct entity in Morocco, predominantly driven by modifiable behavioral risk factors, particularly cigarette smoking and cannabis use. Our findings confirm a characteristic coronary phenotype marked by predominantly single-vessel disease, frequent involvement of the left anterior descending artery, and a generally favorable in-hospital prognosis, although acute complications such as left ventricular systolic dysfunction and cardiogenic shock remain clinically significant.

The notable proportion of MINOCA underscores the heterogeneity of underlying mechanisms and the need for structured diagnostic pathways incorporating advanced imaging when available. Targeted prevention strategies - including aggressive tobacco and cannabis cessation programs, optimization of acute STEMI care, and age-adapted secondary prevention - are essential to reduce the burden of premature myocardial infarction and improve long-term cardiovascular outcomes in this high-risk population.
